# Density-based parallel skin lesion border detection with webCL

**DOI:** 10.1186/1471-2105-16-S13-S5

**Published:** 2015-09-25

**Authors:** James Lemon, Sinan Kockara, Tansel Halic, Mutlu Mete

**Affiliations:** 1 Department of Computer Science, University of Central Arkansas, Conway, AR, USA; 2 Department of Computer Science and Information Systems, Texas A&M University-Commerce, Commerce, TX, USA

## Abstract

**Background:**

Dermoscopy is a highly effective and noninvasive imaging technique used in diagnosis of melanoma and other pigmented skin lesions. Many aspects of the lesion under consideration are defined in relation to the lesion border. This makes border detection one of the most important steps in dermoscopic image analysis. In current practice, dermatologists often delineate borders through a hand drawn representation based upon visual inspection. Due to the subjective nature of this technique, intra- and inter-observer variations are common. Because of this, the automated assessment of lesion borders in dermoscopic images has become an important area of study.

**Methods:**

Fast density based skin lesion border detection method has been implemented in parallel with a new parallel technology called WebCL. WebCL utilizes client side computing capabilities to use available hardware resources such as multi cores and GPUs. Developed WebCL-parallel density based skin lesion border detection method runs efficiently from internet browsers.

**Results:**

Previous research indicates that one of the highest accuracy rates can be achieved using density based clustering techniques for skin lesion border detection. While these algorithms do have unfavorable time complexities, this effect could be mitigated when implemented in parallel. In this study, density based clustering technique for skin lesion border detection is parallelized and redesigned to run very efficiently on the heterogeneous platforms (e.g. tablets, SmartPhones, multi-core CPUs, GPUs, and fully-integrated Accelerated Processing Units) by transforming the technique into a series of independent concurrent operations. Heterogeneous computing is adopted to support accessibility, portability and multi-device use in the clinical settings. For this, we used WebCL, an emerging technology that enables a HTML5 Web browser to execute code in parallel for heterogeneous platforms. We depicted WebCL and our parallel algorithm design. In addition, we tested parallel code on 100 dermoscopy images and showed the execution speedups with respect to the serial version. Results indicate that parallel (WebCL) version and serial version of density based lesion border detection methods generate the same accuracy rates for 100 dermoscopy images, in which mean of border error is 6.94%, mean of recall is 76.66%, and mean of precision is 99.29% respectively. Moreover, WebCL version's speedup factor for 100 dermoscopy images' lesion border detection averages around ~491.2.

**Conclusions:**

When large amount of high resolution dermoscopy images considered in a usual clinical setting along with the critical importance of early detection and diagnosis of melanoma before metastasis, the importance of fast processing dermoscopy images become obvious. In this paper, we introduce WebCL and the use of it for biomedical image processing applications. WebCL is a javascript binding of OpenCL, which takes advantage of GPU computing from a web browser. Therefore, WebCL parallel version of density based skin lesion border detection introduced in this study can supplement expert dermatologist, and aid them in early diagnosis of skin lesions. While WebCL is currently an emerging technology, a full adoption of WebCL into the HTML5 standard would allow for this implementation to run on a very large set of hardware and software systems. WebCL takes full advantage of parallel computational resources including multi-cores and GPUs on a local machine, and allows for compiled code to run directly from the Web Browser.

## Background

Dermoscopy is a prevalent method used by dermatologists in the diagnosis of melanoma and other pigmented skin lesions. Dermatologists use a handy, high-resolution imaging tool called dermatoscope to take dermatoscopic images. Dermoscopy is now a well-established diagnostic tool to improve the clinical recognition of a broad spectrum of various skin disorders. Skin cancer detection is the most important indication of dermoscopy. There is evidence that the use of dermoscopy has increased the accuracy of diagnosis [1]. Carli et al. (2004) [1] showed that the examination of a pigmented skin lesion including melanoma using dermoscopy allows physicians to realize morphologic features which are otherwise not visible to the naked eye. This in turn, comparing to conventional non-dermoscopic examination, allows physicians to reach a more reliable diagnosis of skin lesions. Thus, recent melanoma guidelines promote the use of dermoscopy in skin cancer screening and diagnosis [2].

Skin cancer is the most common form of cancer in the US and over 3.5 million cases are diagnosed annually [2]. The deadliest form of skin cancer is melanoma. Melanoma is a malignancy of melanocytes which are special cells in the skin located under the outer surface epidermis. 15% of melanoma cases are fatal [3,4]. Women at 25-29 years of age are the most-commonly affected group [5]. Although melanoma accounts for only 4% of all skin cancers [6], it is the cause of 75% of skin-cancer-related deaths [1]. Even with the help of dermoscopy, 70% of melanoma claims are still a false-negative diagnosis [7]. Melanoma rates are rising amongst all demographics [8]. With early detection, melanoma can often be cured with a simple excision operation.

Dermoscopy is a set of techniques for optical magnification of a region-of-interest on skin which makes subsurface structures more visible than traditionally photographic techniques [9]. The procedure measures many properties of a skin lesion, such as color, size, symmetry, border, and change over time. The odds of successful diagnosis between naked eye examination and Dermoscopy are 15-6 [10]. Even when dermoscopic images examined by an expert, diagnosis rates are not completely accurate.

An expert system capable of processing dermoscopic images could provide an additional diagnosis tool to aid dermatologist. In many common manual methods of examining photographs of lesions, a border is drawn by a dermatologist, and this border is 'subjectively' analyzed by dermatologist to diagnosis if the lesion is malignant melanoma or melanocytic. Similarly, automated systems designed to processes dermoscopic images usually start with automatic border detection before examining the lesions for the features with diagnostic importance such as color, symmetry, etc. [11]. There are many methods for detecting the border of a lesion. The blue color channel is typically examined because empirical evidence suggests it provides the most accurate results [12]. Reader is referred to [13] for details on analysis of color models and color channels on biomedical image processing.

Border detection is usually the first stage of analysis of dermoscopic images. Our implementation presented here automatically delineates the lesion border by using density based clustering technique. In order to get a clear definition of the lesion, some preprocessing, such as color space transformations, contrast enhancement, and artifacts removal are typically applied to the image [14]. Following this pre-processing, a partitioning of the image occurs in a process known as segmentation. These disjoint regions are then examined by computer algorithms and scanned for lesion data, and combined to detect the border of the entire lesion.

According to Celebi et al. [15] automated skin lesion border detection can be divided into four sections: pre-processing, segmentation, post-processing, and evaluation. The pre-processing step involves color space transformations [16], contrast enhancement [17] and artifacts removal [18]. The segmentation step involves partitioning of an image into disjoint regions [19]. The post-processing is used to obtain the lesion border [20]. The evaluation involves the assessment of the border detection results by a dermatologist. At the first stage of dermoscopy image analysis, border detection is usually applied [21] to detect other features more accurately. An active contour model is also introduced to detect skin lesion borders in dermoscopy images [22]. Many other approaches have been applied to dermoscopy images for accurately segmenting the melanocytic skin lesions. Color histogram thresholding is one of them [23,21]. In Peruch et al. [23] in addition to thresholding, researchers incorporate cognitive process of dermatologists for accurate melanocytic skin lesion segmentation.

Density based clustering algorithms identify regions of high data density, and require a definition of how dense the data should be [24]. Density based spatial clustering of applications with noise, or DBSCAN [24], as its name indicates, is a prominent density based clustering method. It is used for spatial data with noise and has the advantages of being able to find irregularly shaped clusters. DBSCAN also has a sense of border and noise data, and requires minimal knowledge of dataset [25]. It requires two inputs: minimum points, a measurement of how many points need to be grouped; and epsilon (ε), a measurement of how close the points need to be grouped. In the context of this study, points refer to pixels. DBSCAN has many applications, including Internet traffic classification; war-game frontline prediction; and facial recognition [25-27].

**Figure 1:**
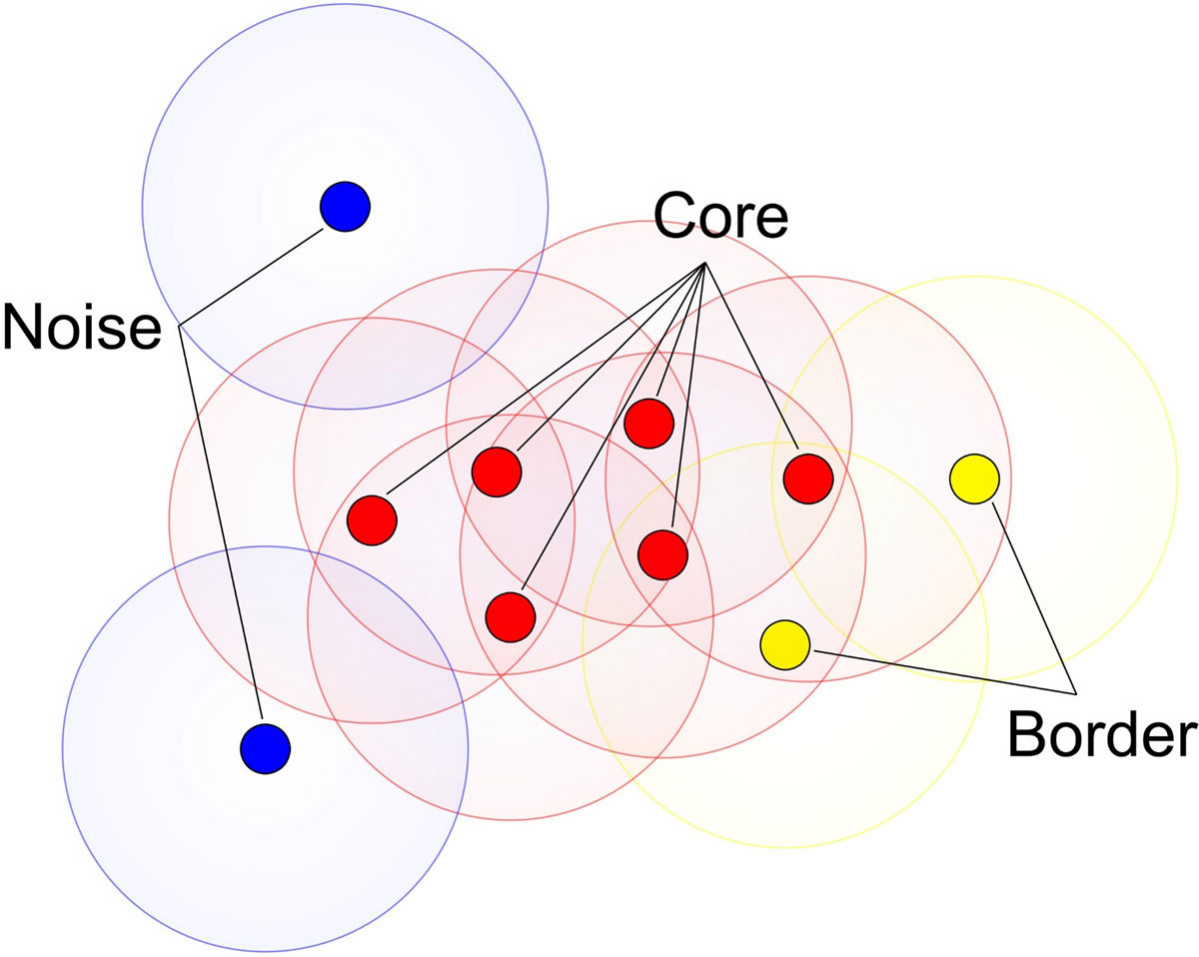
**Graphically describes the different node definitions of Density based scanning**. In the context of dermoscopy images nodes (either they are core, border or noise nodes) are referring to pixels.

**Figure 2:**
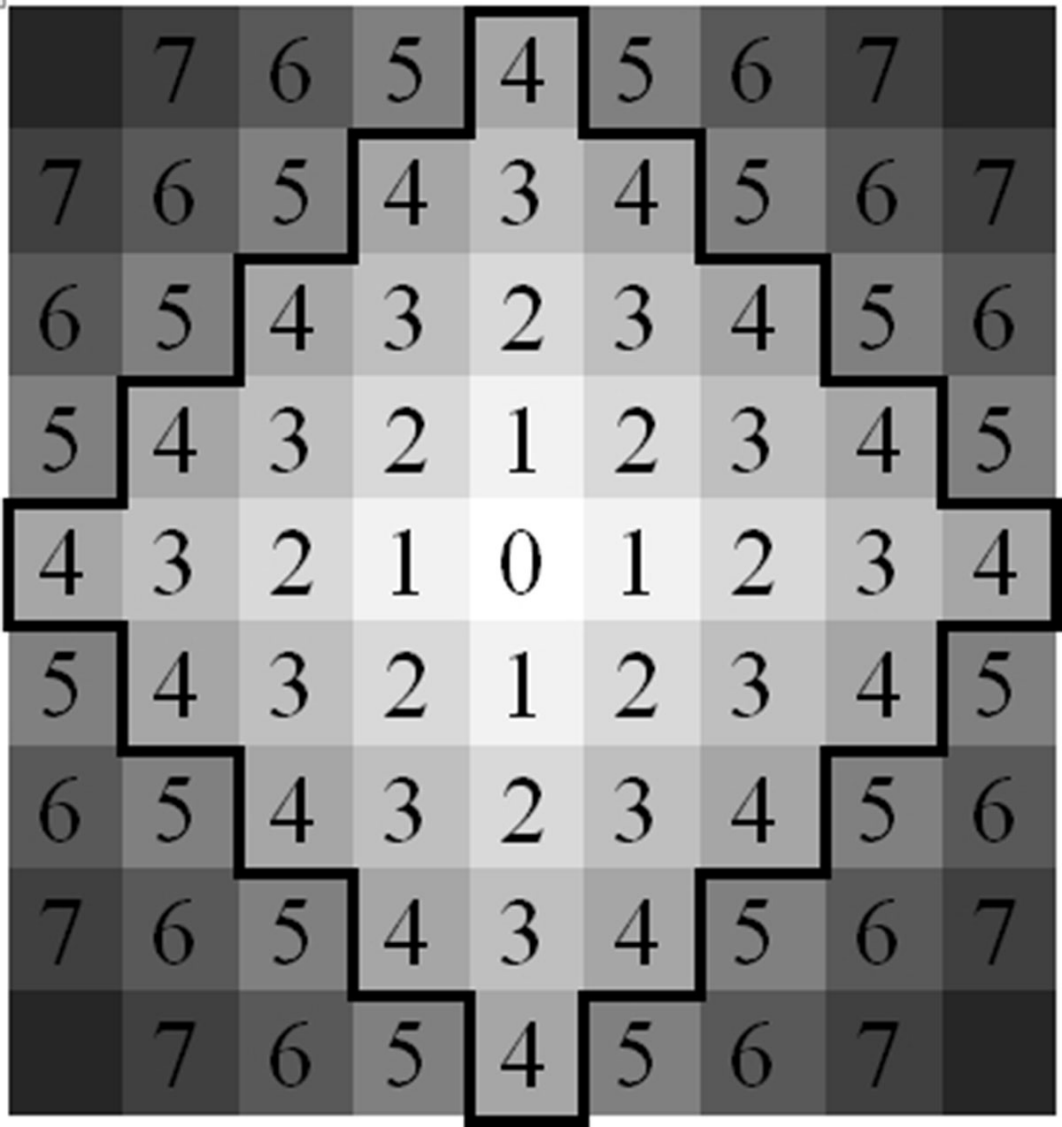
**Manhattan region of radius 4**. Each number is the Manhattan distance from the center point 0.

In DBSCAN, a cluster is a group of points that the number of points is equal to or greater than the minimum number of points (MinPts) in certain neighborhood of core points. Different point (node) definitions of DBSCAN are illustrated in Figure 1. The core point is that the neighborhood of a given radius (ε) has to contain at least a minimum number of points (MinPts), i.e., the density in the neighborhood should exceed pre-defined threshold (MinPts). The definition of a neighborhood is determined by the choice of a distance function for two points p and q, denoted by dist(p,q). For instance, when the Manhattan distance is used in 2D space, the shape of the neighborhood would be rectangular (See Figure 2). Note that DBSCAN works with any distance function so that an appropriate function can be designed for some other specific applications. DBSCAN is significantly more effective in discovering clusters of arbitrary shapes. It was successfully used for synthetic dataset as well as earth science, and protein dataset [28-30]. Once the two parameters ε and MinPts are defined, DBSCAN starts to cluster data points (e.g. pixels for images) from an arbitrary point. If the neighborhood is sparsely populated, i.e. it has fewer than MinPts points in the region query, then that point is labelled as a noise. Points that are causing the cluster to grow called border points. In the context of this paper, terms "node" and "point" are used interchangeably with pixel. Pseudocode of the DBSCAN is given in Algorithm 1.

Algorithm 1 - DBSCAN

Input:

D = Set of all Points

ε = Max distance between nodes

MinPts = Density of nodes needed

Procedure **DBSCAN**(D, ε, MinPts)

**Foreach **unvisited point P in dataset D

P is visited

NeighborPoints = regionQuery(P, ε)

**If(**sizeof(neighborpoints) < MinPts)

P is noise

Else

P = next cluster

ExpandCluster(P, NeighborPoints)

In our previous study we introduced computationally more efficient version of DBSCAN [31] for the purpose of automatic border detection. In that version, we removed redundant computations that exist in DBSCAN by only expanding a cluster around a border region since cluster expands from borders. It was proven in [31] that this approach is computationally more efficient than the traditional DBSCAN.

Even computationally improved version of DBSCAN [31] requires a lot of time for very large datasets. The need for speeding up DBSCAN is better understood when considering high resolution dermoscopic images, which consist of millions of nodes (pixels). Even improved version of DBSCAN given in [31] takes some time for generating results. Reader is referred to Table 1 for the timing of density based skin lesion border detection method's serial version for different size dermoscopy images. Not only image size but also complexity and irregularity of the skin lesion also reduce the performance. Thus, in order to benefiting available ubiquitous high performance computing hardware resources found in today's computers, we implement our density based skin lesion border detection method in a high performance parallel computing model called WebCL. WebCL provides a significant speedup and to provide a high level of portability for our case of dermoscopic image processing.

**Table 1:** Serial vs

Image Resolution	Pixel Count for the lesion	C++ Eps 3 Minpts	WebCL Eps 3 Minpts 4	Speedup Factor
629 × 405	30234	112	3.664	30.56769

749 × 497	36726	161	3.429	46.95246

624 × 425	37648	151	3.383	44.63494

577 × 397	40267	199	3.664	54.31223

635 × 418	50501	313	3.956	79.12032

1090 × 728	50708	307	3.395	90.4271

605 × 419	57556	407	3.195	127.3865

627 × 420	59535	433	3.447	125.6165

744 × 499	60072	435	3.471	125.3241

635 × 421	60102	439	3.759	116.7864

1024 × 684	67909	568	5.693	99.77165

1322 × 875	68961	584	15.029	38.85821

638 × 426	70022	597	4.733	126.1356

755 × 503	71711	622	3.306	188.1428

1076 × 716	78825	759	6.334	119.8295

1024 × 684	79388	774	5.122	151.1128

1024 × 684	79824	779	7.114	109.5024

942 × 629	79998	788	5.97	131.9933

1322 × 875	80352	793	14.475	54.78411

1024 × 684	82274	832	5.335	155.9513

754 × 500	82472	826	3.499	236.0674

756 × 499	84043	859	3.489	246.2024

756 × 497	86201	903	3.459	261.0581

1024 × 684	91591	1028	6.778	151.6672

1024 × 684	95193	1109	5.513	201.1609

1024 × 684	99547	1217	5.643	215.6654

756 × 494	101558	1256	3.344	375.5981

738 × 494	102581	1282	3.323	385.796

756 × 496	103667	1309	3.163	413.8476

1024 × 684	104865	1347	7.164	188.0235

1024 × 684	111114	1512	7.029	215.1088

1024 × 684	121670	1816	6.879	263.9919

1076 × 716	124107	1880	6.479	290.1682

756 × 497	124623	1893	3.243	583.7188

1024 × 684	126503	1954	6.123	319.1246

1024 × 684	130883	2084	6	347.3333

1076 × 716	136201	2273	6.513	348.9943

756 × 495	136977	2295	3.211	714.7306

1024 × 684	137177	2308	6.72	343.4524

1024 × 684	138373	2347	5.422	432.8661

1076 × 716	138756	2356	6.291	374.5033

1024 × 684	141824	2442	6.429	379.8413

1024 × 684	142650	2503	5.303	471.997

1349 × 900	143189	2518	14.124	178.2781

1024 × 684	144278	2557	5.625	454.5778

1024 × 684	150913	2777	6.368	436.0867

1024 × 684	151001	2807	5.238	535.8916

1017 × 683	151703	2826	7.551	374.2551

1076 × 716	152530	2843	6.447	440.9803

1076 × 716	152932	2863	6.591	434.3802

1076 × 716	153182	2869	6.466	443.7055

1076 × 716	154154	2905	6.466	449.2731

1024 × 684	157797	3028	6.463	468.5131

1024 × 684	160899	3167	5.695	556.1018

1024 × 684	161260	3197	5.343	598.353

1076 × 716	164723	3317	6.377	520.1505

1024 × 684	165204	3332	5.561	599.1728

1024 × 684	165366	3373	6.313	534.2943

1076 × 716	166850	3397	6.379	532.5286

1024 × 684	171738	3613	6.945	520.2304

1024 × 684	172285	3617	6.008	602.0306

1024 × 684	179413	3972	5.822	682.2398

1024 × 684	185213	4233	6.566	644.6847

1873 × 1225	185675	4242	28.134	150.7784

1024 × 684	196606	4732	5.432	871.134

1089 × 730	196951	4738	8.677	546.0413

1024 × 684	199503	4856	5.572	871.5004

1024 × 684	200329	4929	6.893	715.0733

1256 × 825	202803	5067	12.61	401.8239

1024 × 684	204368	5128	5.653	907.129

1024 × 684	205127	5157	6.771	761.6305

1024 × 684	206826	5255	5.171	1016.244

954 × 634	208317	5335	7.406	720.3619

1024 × 684	210117	5397	5.636	957.594

1897 × 1267	210989	5472	30.121	181.6673

1024 × 684	213697	5610	5.767	972.7761

1024 × 684	214272	5654	6.746	838.1263

1350 × 903	222613	6076	15.258	398.2173

1024 × 684	226198	6263	5.756	1088.082

1076 × 716	229012	6409	6.38	1004.545

1828 × 1216	239035	7026	28.852	243.5186

1891 × 1261	249382	7653	29.352	260.7318

1024 × 684	249704	7614	5.89	1292.699

1024 × 684	259701	8222	5.989	1372.85

1329 × 909	264550	8564	15.001	570.8953

1149 × 767	284292	9920	9.168	1082.024

1913 × 1280	296438	10633	29.33	362.5298

1389 × 929	301804	11055	11.028	1002.448

2469 × 1602	370476	16793	51.145	328.341

1819 × 1213	415339	21219	27.538	770.5353

1881 × 1260	452778	25159	30.992	811.7901

1813 × 1217	508230	31735	26.739	1186.843

1879 × 1261	577752	41069	30.444	1349.001

1915 × 1256	636255	49630	25.341	1958.486

1849 × 1233	698408	60037	29.487	2036.05

1024 × 684	212484	5540	5.672	976.727

1024 × 684	92093	1041	6.771	153.743

1352 × 899	107441	1415	7.245	195.307

1867 × 1266	328997	13629	13.984	974.613

635 × 419	9424	10	3.123	3.2

WebCL parallel version: Speedup factors of 100 dermoscopy images.

## WebCL

This section summarizes WebCL and driving force behind WebCL. Computing capability of today's computers has been evolving with introduction of more computational cores in chips rather than faster computational cores. This is because of reaching the physical limits of silicon chip, excessive heat dissipation causing noise and high voltage requirement in increasing clock frequency of integrated circuits. As a result, we use multicore and multiprocessors that consists of many computing chips in one single integrated circuit (e.g. multicore CPUs or GPUs). This trend in chip advancement can also be seen in mobile platforms such as smart phones and tablets. Therefore, the performance improvement in any applications, especially applications with high demand in computation power, can be only realized with the use of parallel computation. Although application parallelism for desktop applications is achievable at some degree for quite a while, up until introduction of WebCL web-based client-side applications were not able to use available parallel hardware resources such as multicores and GPUs at the client-side.

Lack of parallelism creates the main limiting factors for many potential web applications. Web applications work solely on the user device and although the device has enough computing ability, application cannot utilize the underlying multi-core and multiprocessor device (e.g. such as IPhone). This is due to the limitations placed on the current Web browsers' designs and lack of any middleware software layer. But with the recent technology called WebCL, it is now possible to design and develop parallel algorithms that could effectively use the parallel hardware. Specifically, WebCL is a recently introduced (first version released in March 2014) open web based standards for heterogonous parallel computing. As it is an open specification, it has been gaining wide acceptance nationally and internationally. WebCL specification has been accepted and driven by Khronos Group (Khronos is an open consortium funded by major software and hardware companies such as Apple, Google, IBM, NVIDIA, Samsung, Qualcomm, PIXAR, Texas instrument etc.). WebCL mainly introduces a new software middleware layer aims at directly accessing to the parallel hardware within the web browsers.

With WebCL, It is now possible to develop high performance web applications such as data visualization, video processing, 3D gaming, interactive simulations, image processing and segmentation that would not be possible before. The Web applications that can now effectively use these computing capabilities of any mobile devices as tablets (e.g. IPad, Android, and Windows based tablets), smartphones (e.g. IPhone, Windows, Firefox OS based phones etc. ) and also prospective devices that will be available in everyday use such as smart watches, smart glasses, and smart devices in smart homes.

The significance of using WebCL is that it removes any device dependency and provides true platform independence. We can design and develop computation intensive web applications that are accessible by any device regardless of their hardware and software platform. This also enables WebCL application portable, mobile, and future compatible; meaning that the application can adopt computing capability of upcoming devices in the future.

Any algorithm developed with WebCL needs to adopt a single instruction multiple sata (SIMD) approach. In SIMD, there is a single execution task working on its own portion of data called kernel. Therefore, kernel can operate in multiple cores at the same time simultaneously and independently which significantly increases the performance of the application.

WebCL is fast and portable. It takes full advantage of parallel architecture in new computing devices. It currently runs on Firefox, Safari, and Chrome web browsers [32]. It can provide instant high performance computing to a desktop, laptop, a tablet, or a smartphone. Compared to java-script, the most popular language for web-based computation, WebCL is 100x faster [32]. In the future, this technology will be implemented on more mobile phones and tablets, allowing for fast computing to be available anywhere, loaded straight from the internet.

WebCL is a parallel programming environment that also takes advantage of general purpose processing for graphics processing units (GPGPU). A GPU has more transistors than a consumer level CPU, and can be considered a more powerful processor [33]. GPUs also produce more FLOPS/watt than CPUs, making them energy efficient alternatives over CPUs [34]. This makes GPUs find application areas in medicine [35]. GPUs use a SIMD programming paradigm. It computes a kernel, or an algorithm, on a stream, or ordered sequence of the same type of data, in parallel. A kernel operates on an entire stream.

WebCL is a proposed standard, first announced in March 2011, designed to provide higher performance client side computation [36] for web interfaces. WebCL was noted by the NSF Cross-layer Power Optimization and Management workshop for increasing performance, productivity, and portability [37]. WebCL is a set of JavaScript bindings to OpenCL. OpenCL is a high level abstraction that allows for high performance code to run on a large variety of devices [38]. It is an open specification [39]. WebCL is currently in the API definition phase, and has three popular open source implementations; Samsung, Nokia, and Motorola [36]. For our purpose of parallel skin lesion border detection, we use Nokia's WebCL implementation.

A goal of WebCL is to provide a platform for a large variety of high performance web applications to run on multicore devices [40]. To run a WebCL program, a computer needs a modified web browser, OpenCL hardware capable hardware, and OpenCL driver software [40]. WebCL programs start on the JavaScript application level. WebCL kernel is built from source to be optimized on the local device that it will run. Jobs are created by matching a kernel with a datastream, and are executed by queuing the job in the command queue. Jobs on the command queue executes directly on the device. When a job is complete, a JavaScript event may be created to signal its completion, which can be used to process output or as a barrier for tasks that need to be serialized. WebCL has a high interoperability with WebGL, similarly to how OpenCL has high interoperability with OpenGL [32].

Nokia's implementation of WebCL is a Firefox extension. WebCL support can be added to Firefox by installing OpenCL drivers for a local device, and installing Nokia's WebCL add-on. Samsung's WebCL implementation runs on Safari, and is built on top of WebKit. It must be installed from source code, and also relays on OpenCL drivers for a local device. The two implementations agree upon a single API, which is currently a working draft. Both of these implementations are capable of supporting applications in their current state. For our purpose, we use Nokia's Firefox Add-on for WebCL due to ease of deployment, portability across platforms, and access to developer add-ons native to Firefox. Although WebCL has many benefits such as accessibility, portability, and performance, it requires significant design and development time, and more importantly expertise in parallel computation and web application development. This is due to the fact that WebCL can only benefit from the simultaneously execution of application tasks on multiple hardware computation cores. For any application a new novel parallel algorithm needs to be developed designated for WebCL execution scheme and parallel hardware.

**Figure 3:**
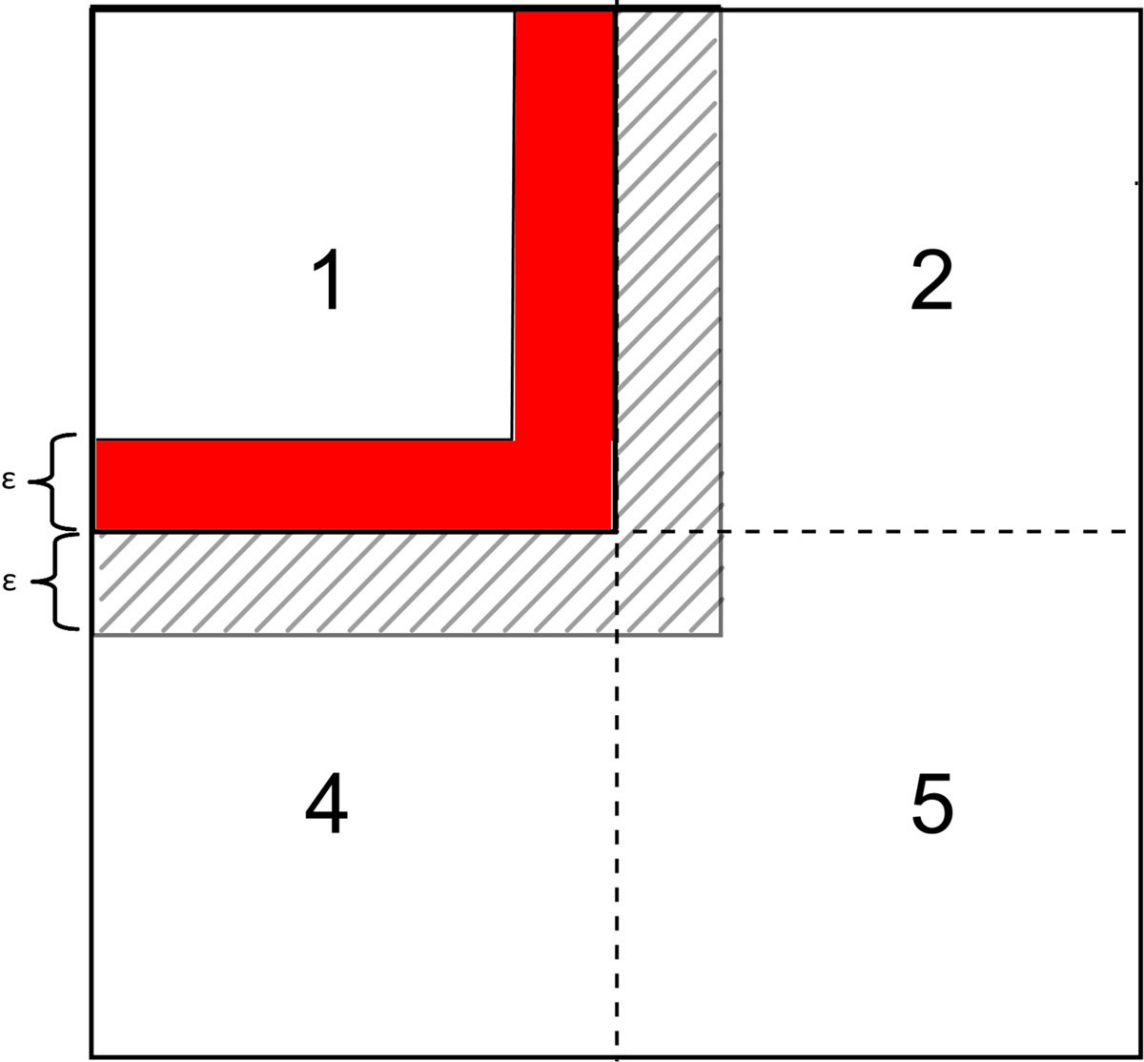
**Each shingle has a redundant zone with the shingle to its immediate right, the shingle immediately below it, and the shingle to its bottom-right**. This allows for partition merging using the transitive property.

Our implementation uses Manhattan Distance to calculate the distance between two pixels. It has two large advantages leading towards a small computational complexity: it maps easily to integer space which performs quickly on a GPU, and it maps naturally onto an image [41]. Manhattan Distance is the change in × plus the change in Y. Distance based calculations are very common in density based clustering. Figure 3 illustrates a sample radius 4 Manhattan region with the region's center being 0. Next section introduces WebCL implementation details of the density based skin lesion border detection method.

## Methodology

**Figure 4:**
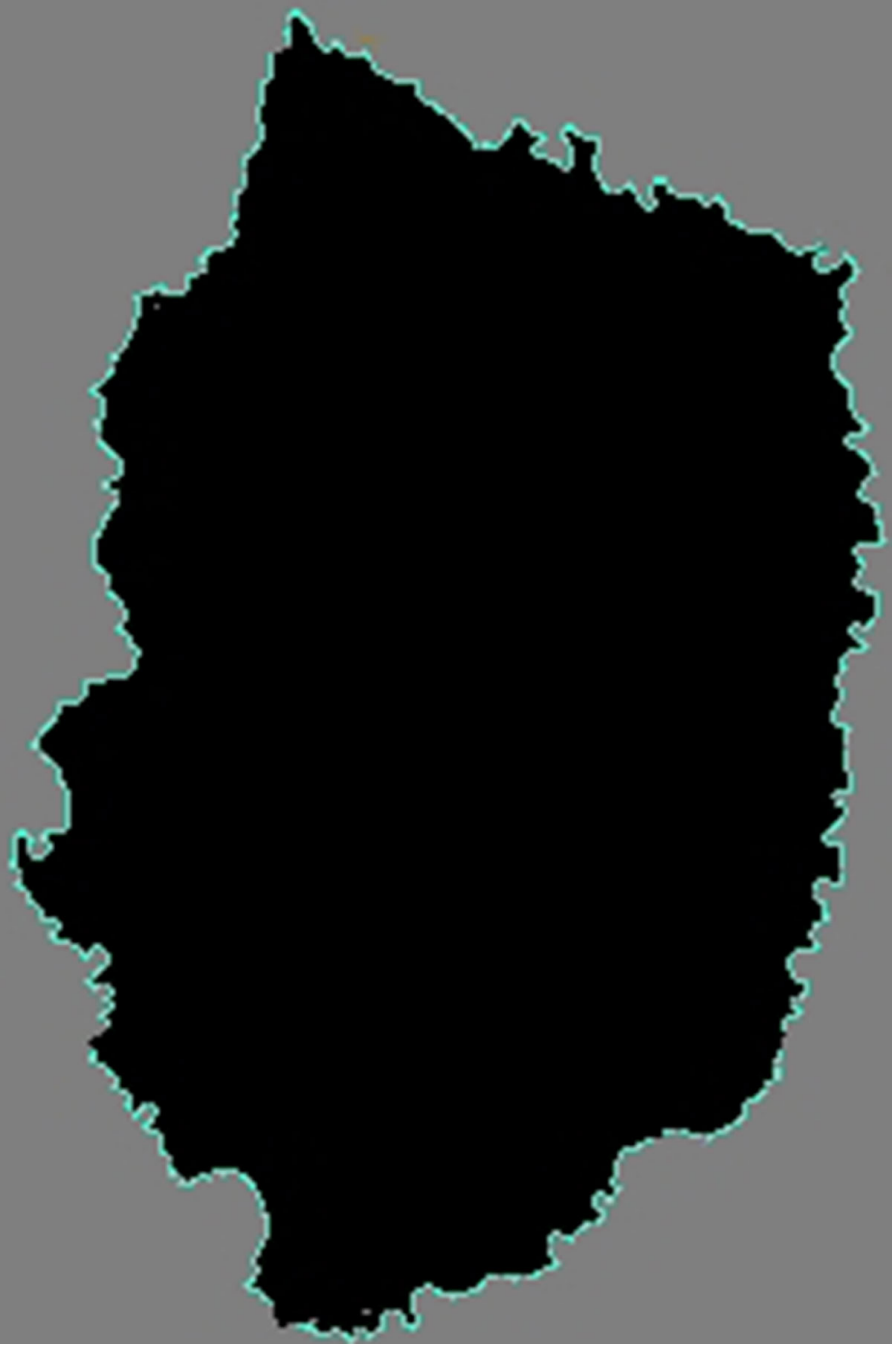
**Automatically generated border of skin lesion displayed on an HTML5 canvas created by a WebCL kernel**.

To take full advantage of the acceleration available through WebCL, our previous density based skin lesion border detection method was implemented in data-parallel. Nodes are divided into geographic regions, with some redundancy in the area covered in order to limit the cross thread communication requirements. Each node is assigned to a thread, creating up to millions of threads, and the distance is calculated between nodes in the same previously created geographic regions. Using the transitive property, the output of each thread is combined using a tree reduction algorithm. A border is detected by noticing a drop in density amongst the nodes. After the image has been scanned, a line is drawn displaying the border of the lesion. After the image has been processed, it is drawn in an HTML5 canvas with border regions represented in a color that contrasts with the lesion image, thus delivering the border. This is illustrated in Figure 4.

### C++ Implementation

A serial version of density based skin lesion border detection method was implemented in C++. Following that, a parallel WebCL version was designed and implemented, taking full advantage of the speedups made available by the WebCL framework. The serial C++ implementation reads a dermoscopy image, and generates every pixel's cluster id along with whether that pixel is border, core, or noise. The serial C++ version of the implementation uses the pseudocode mentioned in Algorithm 1.

### WebCL implementation

The WebCL implementation accepts a dermoscopy image file as an input. The input file is divided into sizes capable of being stored inside the GPU. More specifically, a dermoscopy image partitioned on to a smaller pieces to be concurrently executed on the GPUs. For effective image partitioning available memory spaces in the GPUs are considered. Nokia's Firefox implementation currently has a tight memory limit. Thus, we had to incorporate this limitation along with the available memory space in the GPUs. Each image partition is then scanned using the parallel implementation of density based skin lesion border detection (see Algorithm 2). The output of each scan (Region Query in Algorithm 2) is merged in to a global memory in the GPU such a way as to combine clusters span that across multiple partitions/borders. How image data is partitioned and how borders on different image partitions are merged are explained in the following section. More specifically, next section explains how merge operation is designed and implemented. Due to the nature of independent concurrent threads running the image partitions, the same clusters falling in to different partitions may be labelled differently. In order to eliminate that problem after parallel threads executions on different image partitions, we need an additional operation called merging.

Algorithm 2 - Parallel version of DBSCAN for each image partition using density based clustering to find the data density around a certain location

Input:

N = Contains a location in an.Image Partition

Partition.Image = A 2D array, each location may be a node

Eps = The distance from the center of N to be searched

Output:

Neighbors = A list of all Nodes within Eps distance of N

Procedure **RegionQuery()**

**For each **Location T inside an Image Partition

**If **T is a node

  **If **The distance between N and T <= Eps

    Neighbors.add(T)

### 1) Shingled partitioning

Many devices have memory limitations. Thus, partitioning an image is necessary if the image size is larger than the available memory space. This is better understood when virtual slides are considered. For instance, virtual slides with 10 GB size are quite common. For these very large images, partitioning the image and processing the partitioned image inside the GPU is necessary. Not only that, image partitioning is also important for scalability and portability. A modern day GPU typically can support 2 GB of data. Image partitioning increases the portability, because a device queue can be created to determine a maximum partition size. So that later data can be partitioned and processed in the GPU's device queue. This allows for an application to be optimized for high-end hardware, while still running on lower end or mobile hardware. So, when data is partitioned dynamically according to the available resources and data size, the application can also be dynamically scaled up or down. Algorithm 3 summarizes image partitioning data structure. Xoffset and Yoffset in Algorithm 3 are determined dynamically with an image size and the available resources in the GPU.

Algorithm 3 - Image Partitioning Data Structure

DataStrcture **Partition**

Image[][] = 2D array, each position (pixel) is a node

Xoffset = Where this partition starts on the × axis of the whole image

Yoffset = Where this partition starts on the Y axis of the whole image

Because density based skin lesion border detection uses the density of a point's (a pixel) ε-neighborhood, true partitioning, wherein any given pixel must reside in one and only one partition, would not be satisfied. This is due to the fact that for a point to be properly categorized as a core node, a border node, or a noise node along with a proper cluster ID, the algorithm must be able to examine that point's neighborhood within a radius of ε. This is not a problem for the points falling inside the partition. However, there is a need for a special care for the points (pixels) lying on the edge of a partition (e.g. a neighbor of another partition). This special care is handled by examining the edge points with a large portion of their ε-neighborhood missing (residing in the adjacent partition). More specifically, to deal with the edges of a partition, it becomes necessary to have some redundancy in the border regions of partitions. We call these redundant regions together with the partition that owns the redundant region as shingles. More specifically, in our implementation, each shingle is composed of a core partition plus a lap region which overlays a portion of the core regions of the shingles to its right and bottom. For instance, core partition of partition 1 with the length of ε is illustrated as a red region in Figure 3. The lap region is 2ε+1 pixels, as needed to accurately measure the density of a point residing near a border.

For instance, Figure 3 illustrates shingle 1 as partition 1 along with the redundant zone (lap region) which is illustrated as a shaded area. Notice that, width of that shaded region is selected as ε for not to miss ε-neighbors of the edge points in the red area (core partition). So that, clusters in partition 1 including edge points/nodes ensured that they will have the same cluster IDs with partitions 2, 4, and 5. This happens when partition 1's edge points' ε-neighbor region query determines that points in partitions 2, 4, and 5 fall in to the same cluster.

Serial image partitioning implementation is a simple O(P) operation where P is the number of partitions. In our parallel implementation, partitioning is also done in concurrently. So that, each thread creates its own unique partition. Uniqueness is guaranteed by unique thread offset which is calculated from thread IDs. Creating partitioning concurrently in different threads reduces computation complexity for partitioning to O(1). The × and y offsets along with the number of rows and columns of partitions are calculated from the core partition size, while the total partition height and width are 2ε+1 pixels larger than their respective core sizes due to the inclusion of the lap region. Pseudocode for image partitioning is given in Algorithm 4. Once image is partitioned then next stage is processing these partitions concurrently with density based skin lesion border detection method.

Algorithm 4 - Method for creating list of partitions

Input:

Partitionwidth = width of a partition

Partitionheight = height of a partition

Height = height of image

Width = width of image

Output:

partitionList = List of Partitions

Procedure **createPartitions()**

partsPerRow = width / partitionwidth

PartRows = height / Partitionheight

**For each **row in partsRow

**For each **partition in partsPerRow

  xOffset = indexof partition in Row * PartitionWidth

  yOffset = indexof row in partsRow * Height

  partition = new Part(xOffset, yOffset)

  partitionList.add(partition)

### 2) Partition processing

Partition processing also occurs in parallel inside a WebCL device. Partition processing finds clusters within a single partition. In this stage each pixel of a partition in the image is given a single thread. The thread checks whether the node (pixel) is a core/border/noise node. Then every node in a cluster negotiates a cluster id, agreeing upon the smallest pixel id as a cluster id. The negotiation process is run iteratively until all elements agree. Partition processing summarized in Algorithm 5 and 6 accordingly.

**Algorithm 5 - Partition processing. It is performed for every pixel in parallel. This scans for the minimum neighbor ID**.

Input:

Partition = A partition to process, a global variable

Output:

NodeList = A list of all nodes in a partition

C=an integer value of the cluster ID

Procedure **PartitionProcessing() //**create 1 thread per pixel in parallel

Sum = 0 Sum2 = 1

**While(**Sum !=Sum2)

  Sum2 = Sum

  Sum = 0

  NodeList = Scan(Partition);//scans for minimum cluster ID among ε-neighbors

  **Foreach **Node N in NodeList

  Sum += N.C

**Algorithm 6 - Scan operates on the data stream of an image, segmented and loaded as a partition. The partition is loaded into the constant memory**.

Input:

Partition = A partition to search for clusters in, a global variable

minPts = A measure of minimum cluster density, a global variable

Output:

NodeList = A list of all nodes and an associated integer value C or cluster ID, a global variable.

Procedure **Scan()**//1 thread per pixel in parallel

Pixel n = partition.image[x][y];

**If**(n is a node)//core or noise or border

Neighbors = Region Query// ε-neighbors

**If**(Neighbors.size >= minPts)

  n.C = Smallest C of all Neighbor's Cs

**Else if**(p is within the range of a core node)

  n.C = Smallest C of all Core nodes in n's neighbors

**Figure 5:**
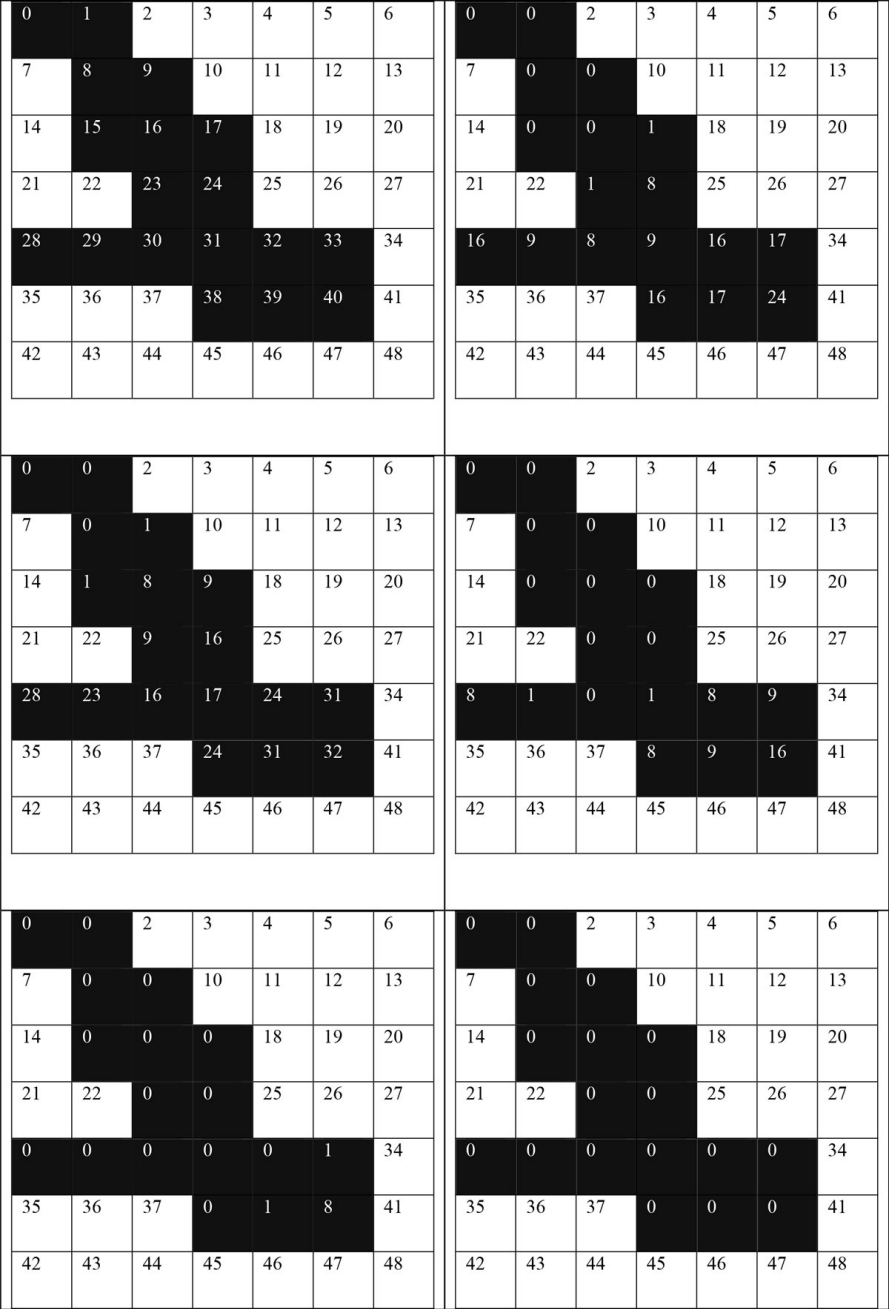
**An exemplary iterative scanning process for eps 2**.

NodeList.add(n);

Figure 5 illustrates an exemplary scanning process step by step for ε=2 and minpts = 2 in a binary image. Initially integers are pixel IDs. In each iteration pixel IDs are changed to the minimum cluster ID of the ε-neighbor. In each iteration, ε=2 neighbors' IDs changed to the minimum cluster ID.

**Figure 6:**
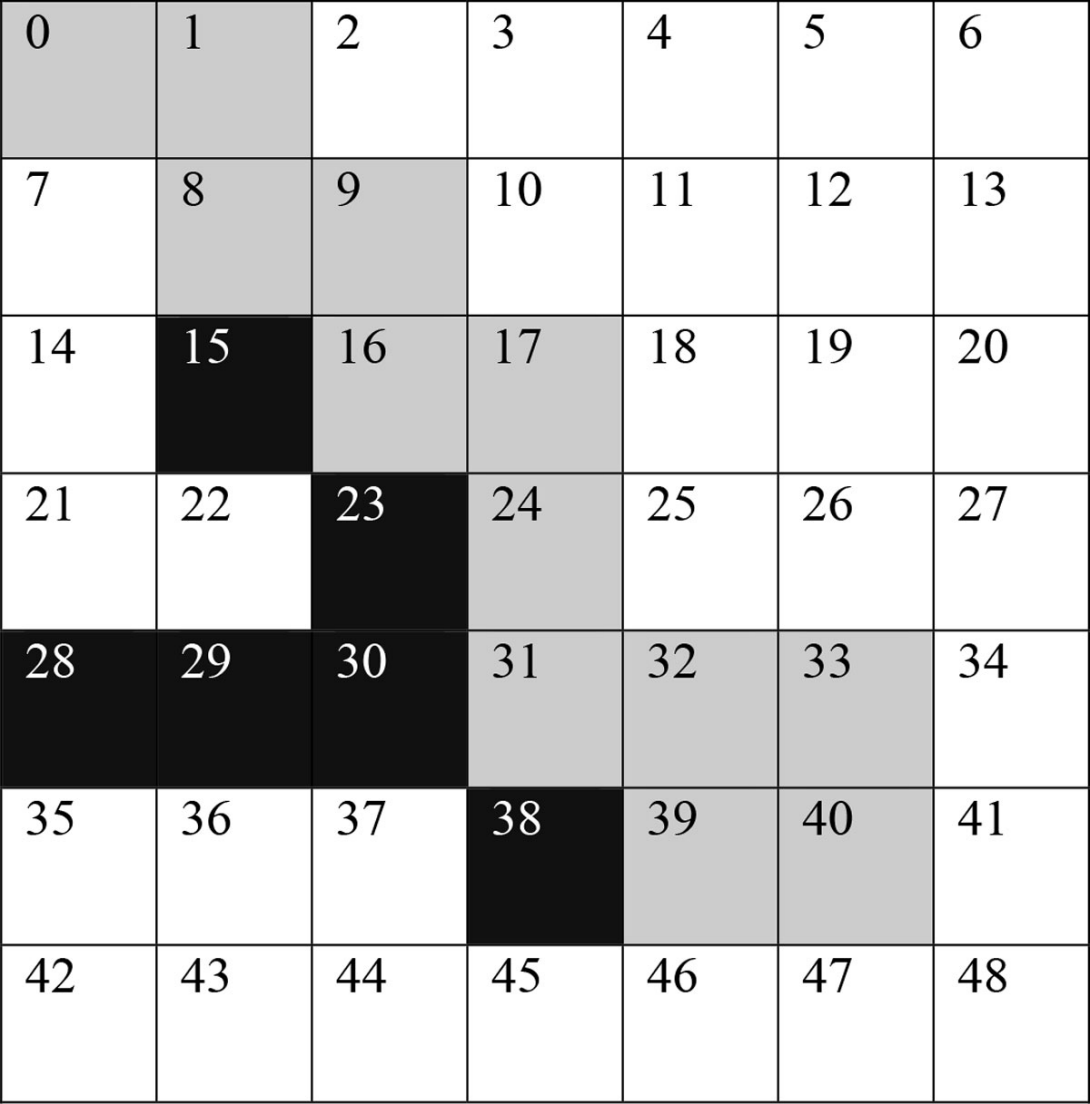
**The longest path, the gray pixels**. If k = longest path + 1 then number of iterations = (k / eps).

**Figure 7:**
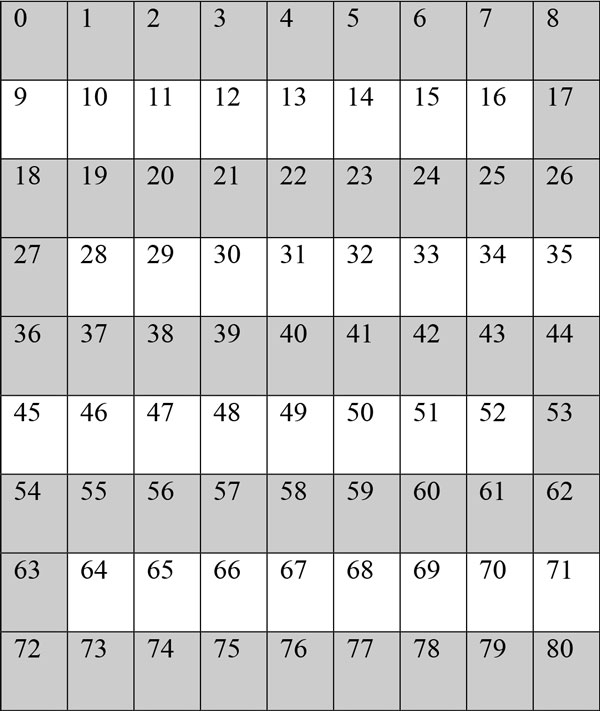
**The worst case scenario: the longest possible path with an ε of 1**. It will require 44 iterations, one for each pixel.

The longest path of the merged region determines how many iterations of scan must be completed before each node in a cluster is associated with the same cluster ID. Figure 6 illustrates the longest path as a grey area for the clustered group of pixels in Figure 5. Figure 7 illustrates the worst case scenario.

### 3) Partition merging

Partition merging works by using the transitive property. Areas of the image that are on the border of a partition are scanned twice. If this node is found in two clusters, than the transitive property demonstrates that the two clusters are actually the same cluster. Nodes clustered in two partitions can be found on the border of a single partition, within 1 ε + 2 of an edge of a partition. This process is completed by merging each partition with a list of all partitions already merged. Algorithms 7 and 8 summarize the merge operation.

**Algorithm 7 - shows the high level process of splitting an image into smaller pieces and combining the output of the smaller pieces**.

Output:

GlobalNodeList - A lis**t **of all nodes in the entire image

Procedure **MergeClusters()**

PartitionList = CreateParititons()

**For each **partition in PartitionList

NodeList = **ParitionProcessing(**partition**)**

**PartitionMerge(**NodeList, GlobalNodeList)

**Algorithm 8 - Partition merge operation: shows how clusters spanning multiple partitions are detected**.

Input:

NodeList - A list of all nodes in a partition

GlobalNodeList - A list of all nodes in the entire image

Procedure **PartitionMerge**()

**For each **node in a cluster in Partition

GlobalNodeList.add(node)

**For each **node in GlobalNodeList Within Eps of a Parition border

**if **two nodes are in the same location

  Merge the clusters associated with each node

**Figure 8:**
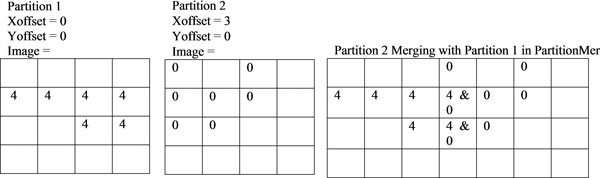
**shows how shingling partition overlapping can be used to identify clusters the span multiple partitions**.

Figure 8 illustrates how partitions processed by different threads are merged in later stages of the computation. In this specific example, regions with cluster IDs 4 and 0 are merged.

### 4) Finding the border

Density based clustering can classify nodes into three separate categories, core, border, and noise. A core node has high neighbor density that means that the node has more than MinPts number of neighbors. A border node is inside the neighborhood of a core node, but it does not have a neighbor density greater than MinPts. A noise node has sparse neighbor density, and is not neighbor with any core nodes. We use these definitions to determine the border of a skin lesion, and use it to handle noise values. After each node is classified, we can use the X, Y data of each node to draw the core and border nodes onto an HTML5 canvas. We draw border nodes using a color with high contrast to the color we draw border nodes with (see Figure 2 for an example). This allows for the border to be easily visible. Algorithm 9 demonstrates the step by step procedure of classifying each node into three categories: core, border, and noise.

Algorithm 9 - Node classification

Input:

GlobalNodeList = A list of all nodes in the image

MinPts = Minimum density to be determines a cluster

Output:

CoreNodeList = A list of nodes that are at the core of a cluster

BorderNodeList = A list of nodes that are at the border of a cluster

NoiseList = A list of nodes that are not in any cluster

Procudure **ClassifyNodes()**

**For each **Node N in GlobalNodeList

ClusterCount = Number of Nodes A where A.C = N.C

**If **ClusterCount < MinPts

  NoiseList.add(N)

**Else if **RegionQuery(N, GlobalNodeList).size < MinPts

  BorderNoeList.add(N)

Else

  CoreNodeList.add(N)

## Results and discussion

Amdahl's law [42] for a maximum theoretical speedup states that an algorithm can be accelerated by the portion of the algorithm that is parallelizable plus the portion of the algorithm that is serial. In the serial version of density based skin lesion border detection, each pixel needs to look up ε^2 ^other pixels to determine density. Drawing the image is done in linear time big O(#pixels). The serial time is ε^2^*#pixels, or O(ε^2^). The speedup achievable according to Amdahl's law is ε / #pixels, or big O(ε). This turns the time complexity from quadratic based on ε and pixel count to a linear based on ε. This assumes that each pixel can be assigned a thread that is run concurrently. Our implementation does not achieve Amdahl's law because of communication overhead. However, the parallel WebCL version of density based skin lesion border detection algorithm achieves an average of around ~491.2 speedup over the serial version on 100 dermoscopy images. See Table 1 for a list of speedup factors for 100 dermoscopy images along with the resolution of each image. While parallel version has obtained considerable speedups, it had exactly the same accuracy ratios of the serial version given in our previous work [31] in which mean of border error is 6.94%, mean of recall is 76.66%, and mean of precision is 99.29% respectively. Schaefer et al. [43] is the first study that uses border error (XOR) measure for dermoscopic image analysis.

To determine that parallel version of the algorithm is generating the same accuracies with the serial version for the same images; we conduct controlled experiments on the same images. Controlled experiments mean that whatever the order of randomly selected pixels in the serial version is, we used the same order for the pixels in the parallel version. With these controlled experiments, we obtained the same accuracy ratios as given in Table 2 for both the serial and parallel versions. Table 2 shows precision, recall, and border error for 100 dermoscopy images. However, both serial and parallel versions of the algorithm each time randomly select the pixels for processing, then results may not have exactly the same accuracies. By the way, discrepancy of accuracies will be unnoticeable (e.g. 99.13089% vs. 99.13091%). This unnoticeable discrepancy is even true for the serial version; when serial version runs on the same image at different times (means that generates different order of randomly selected pixels). Thus, it cannot generate exactly the same results.

**Table 2:** Border error, precision, and recall measures for each image in the dataset.

Img. ID	Border Error	Precision	Recall	Img. ID	Border Error	Precision	Recall
1	8.2%	0.98	0.79	51	5.1%	1.00	0.81

2	8.0%	0.93	0.86	52	6.9%	1.00	0.80

3	4.9%	0.89	0.85	53	7.4%	1.00	0.78

4	6.2%	1.00	0.82	54	1.5%	1.00	0.95

5	5.4%	1.00	0.88	55	4.2%	1.00	0.88

6	4.6%	1.00	0.83	56	14.9%	1.00	0.60

7	3.9%	0.96	0.91	57	9.4%	1.00	0.77

8	3.2%	1.00	0.87	58	5.9%	1.00	0.82

9	3.4%	1.00	0.82	59	4.6%	1.00	0.75

10	2.2%	1.00	0.91	60	2.9%	1.00	0.81

11	0.9%	1.00	0.91	61	6.5%	0.90	0.74

12	6.5%	1.00	0.61	62	5.8%	1.00	0.74

13	10.0%	1.00	0.70	63	5.6%	1.00	0.77

14	14.8%	1.00	0.70	64	2.9%	1.00	0.82

15	5.9%	1.00	0.67	65	2.2%	0.94	0.84

16	6.8%	1.00	0.76	66	8.3%	0.89	0.79

17	6.0%	1.00	0.67	67	6.3%	0.98	0.83

18	4.0%	1.00	0.86	68	3.2%	1.00	0.79

19	6.4%	1.00	0.71	69	2.4%	1.00	0.79

20	8.0%	1.00	0.80	70	4.6%	1.00	0.74

21	8.8%	1.00	0.78	71	8.8%	1.00	0.71

22	12.6%	1.00	0.73	72	3.5%	0.94	0.84

23	8.6%	1.00	0.76	73	1.8%	0.99	0.86

24	9.0%	1.00	0.72	74	2.9%	1.00	0.90

25	5.7%	1.00	0.79	75	5.9%	1.00	0.71

26	33.9%	1.00	0.51	76	9.2%	1.00	0.74

27	9.0%	1.00	0.74	77	3.3%	1.00	0.72

28	8.0%	1.00	0.65	78	13.6%	1.00	0.61

29	10.6%	1.00	0.75	79	10.4%	1.00	0.71

30	11.3%	1.00	0.74	80	6.7%	1.00	0.65

31	9.7%	1.00	0.72	81	1.8%	1.00	0.65

32	10.8%	1.00	0.77	82	7.5%	1.00	0.82

33	3.3%	1.00	0.86	83	9.9%	1.00	0.54

34	4.2%	1.00	0.88	84	3.1%	1.00	0.74

35	2.7%	1.00	0.88	85	6.4%	1.00	0.79

36	6.0%	1.00	0.79	86	7.5%	0.98	0.79

37	4.0%	1.00	0.85	87	7.2%	1.00	0.73

38	8.0%	1.00	0.71	88	5.1%	1.00	0.59

39	3.4%	1.00	0.76	89	5.5%	0.91	0.82

40	3.6%	1.00	0.82	90	17.0%	1.00	0.56

41	8.0%	1.00	0.73	91	8.1%	1.00	0.61

42	3.2%	1.00	0.85	92	4.3%	1.00	0.89

43	7.3%	1.00	0.74	93	1.7%	1.00	0.93

44	17.7%	1.00	0.70	94	14.6%	1.00	0.66

45	3.6%	1.00	0.84	95	3.0%	1.00	0.68

46	5.2%	1.00	0.88	96	7.8%	1.00	0.75

47	2.5%	1.00	0.91	97	21.8%	1.00	0.66

48	3.0%	1.00	0.87	98	4.0%	1.00	0.85

49	10.9%	1.00	0.68	99	11.5%	1.00	0.65

50	12.0%	1.00	0.68	100	3.1%	1.00	0.66

**Figure 9:**
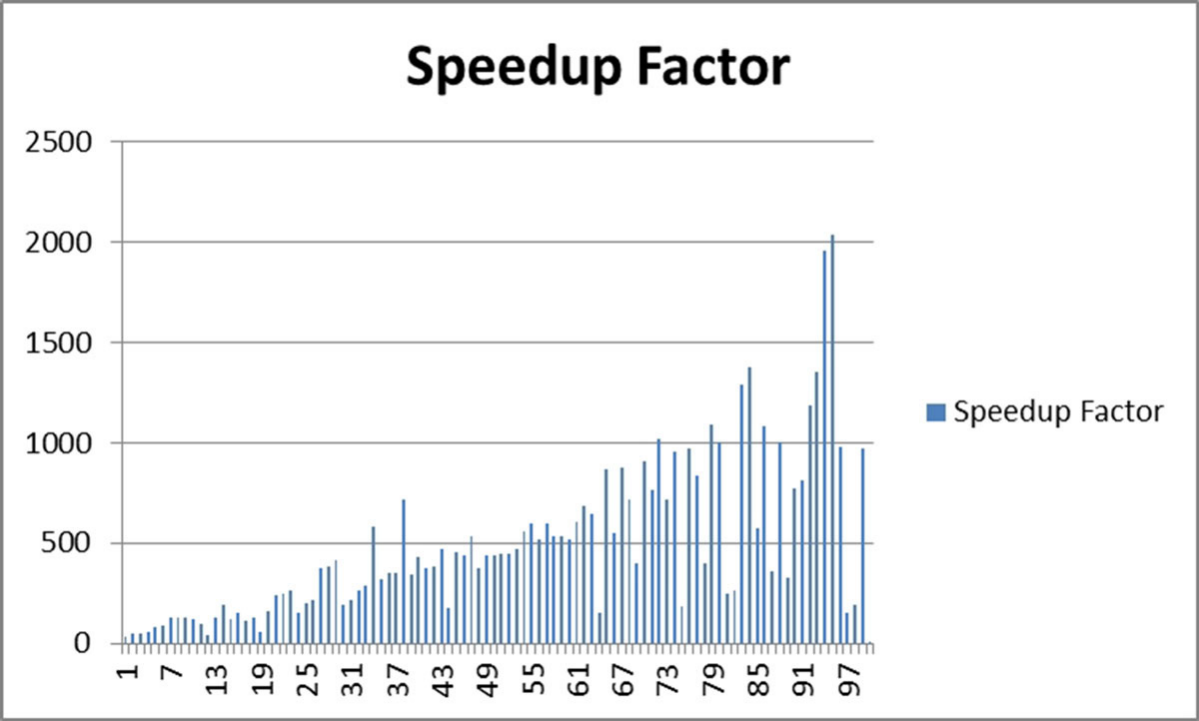
**plots speedup factors of 100 dermoscopy images**.

**Figure 10:**
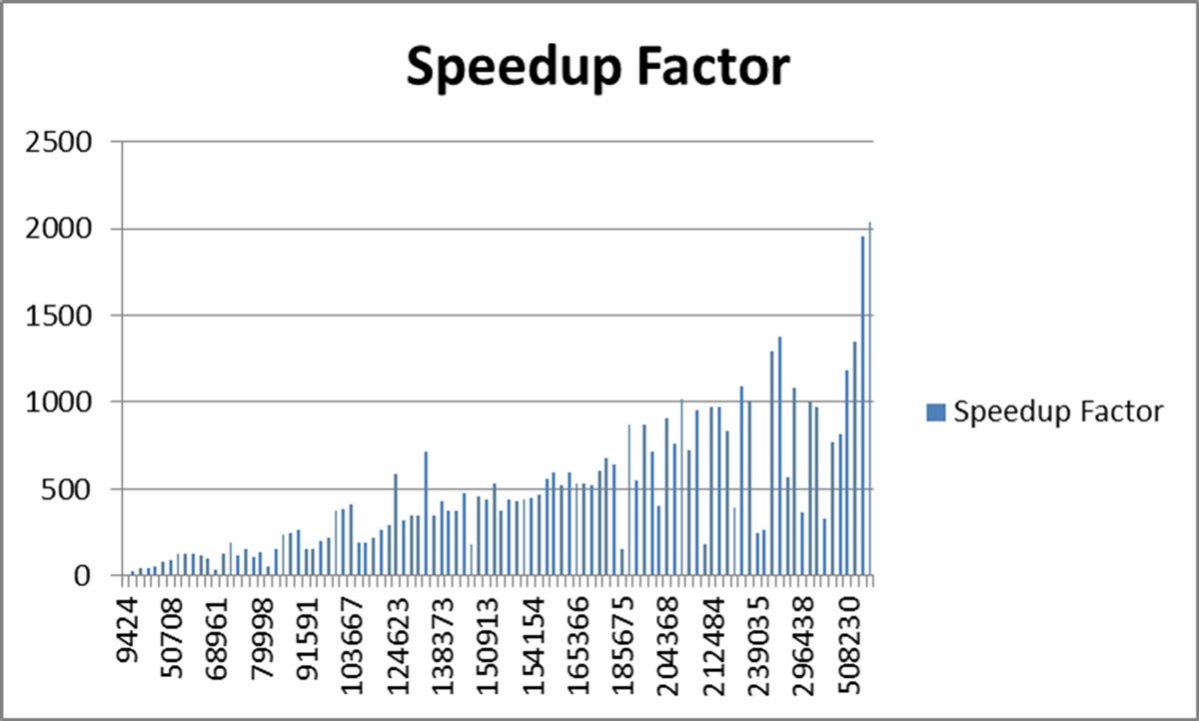
**plots speedup factors of 100 dermoscopy images by the size of the lesion in order**.

Table 1 summarizes all the results obtained for 100 dermoscopy images for both serial and WebCL parallel versions. It also shows speedup factors for each dermoscopy image. Figure 9 plots speedup factors of 100 dermoscopy images with varying resolutions. Figure 10 plots speedup factors of 100 dermoscopy images by the size of the lesion (number of pixels in the lesion) in order.

**Figure 11:**
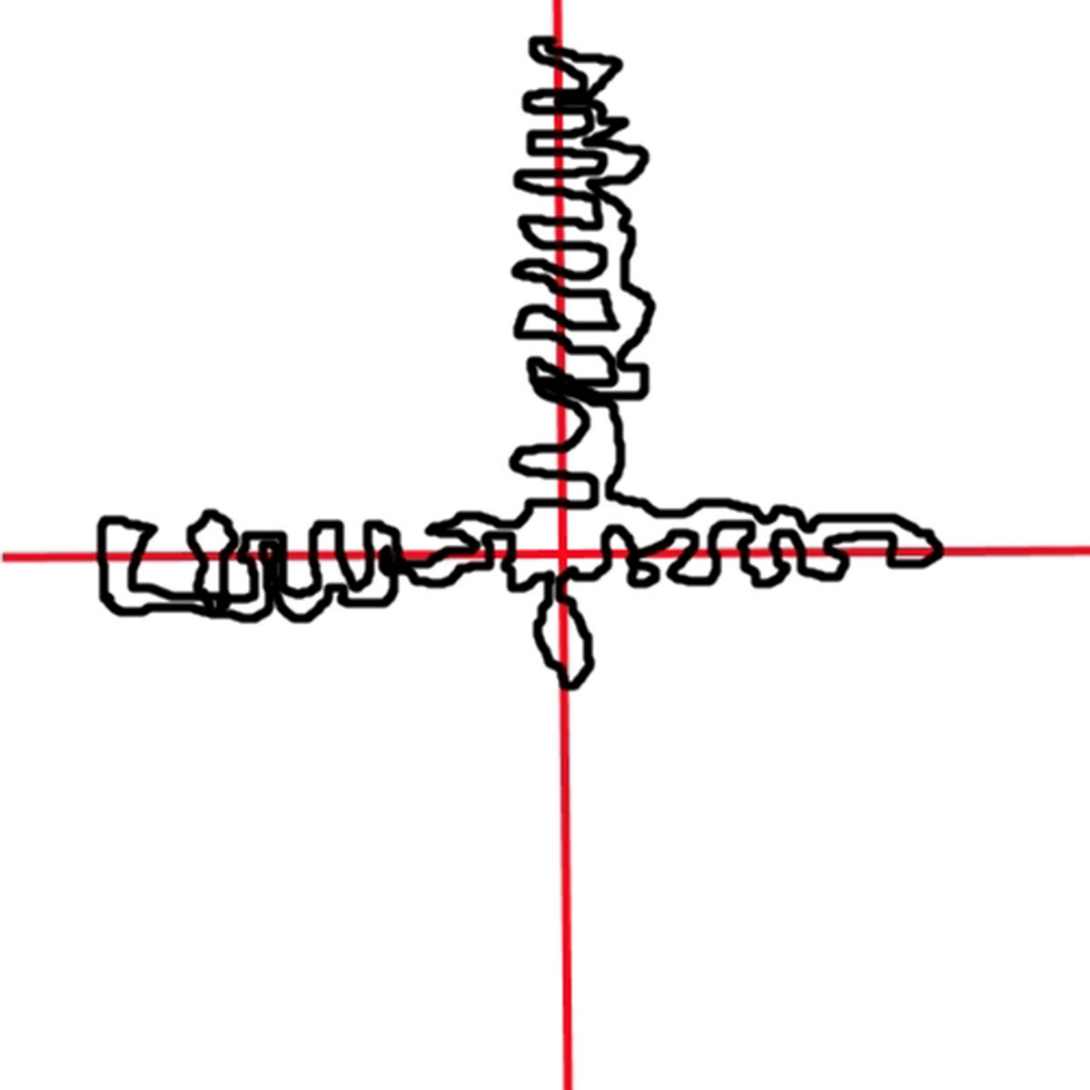
**An exemplary artificial lesion shape (black) with 4 parallel partitions separated by red lines**.

As seen from Figure 10, in some cases even though lesion size is smaller for some images, they have lower speedup factors or vice versa. In some cases; however, lesion size is large but speedup is large too. This is because: the lesion's shape is highly irregular so either causing many region queries or there are many merge operations in the parallel version. Depends on the shape of a lesion, many merge operations may occur even for small size lesions in parallel version. See Figure 11 for an artificially created example. For instance, in this case assume that there are 4 parallel sections which are illustrated by red lines in Figure 11. All of these parallel sections will run concurrently. Thus, each of these concurrent tasks will find too many clusters in their local partitions. However, as can be seen from Figure 11, all of these clusters eventually fall in to the same global cluster. Therefore, image resolution or lesion size may not always be a good indicator for a speedup.

## Conclusions

Using a parallel version of density based skin lesion border detection can provide quick skin lesion boarder detection for dermoscopic images while keeping the accuracy of the serial version. Automated border detection can supplement expert dermatologist, and aid them in diagnosis of melanoma or other pigmented skin lesions. While WebCL is currently an emerging technology, a full adoption of WebCL into the HTML5 standard would allow for this implementation to run on a very large set of hardware and software systems through web browsers. WebCL takes full advantage of parallel computational resources on a local machine, and allows for compiled code to run directly from the Web Browser. This makes it a good candidate for computationally expensive algorithms to be placed in a web browser.

## Competing interests

The authors declare that they have no competing interests.

## Authors' contributions

SK made the overall design of the study. MM provided insights and analysis of the density-based algorithms. JL implemented the WebCL version as a graduate student under SK's supervision. SK developed the general comparison testbed, performed data analysis, algorithm testing, and statistical measurements. JL made benchmarking. TH helped develop design parallel algorithm along with the help of implementing the algorithm in WebCL. JL, SK, and TH contributed to the writing of this manuscript. All of the authors read and approved the manuscript.

## References

[B1] Carli P De Giorgi V Chiarugi A Nardini P Weinstock MA Crocetti E andGiannotti B Addition of dermoscopy to conventional naked-eye examination in melanoma screening: A randomized study Journal of the American Academy of Dermatology 2004 50 5 683 689 10.1016/j.jaad.2003.09.009 15097950

[B2] Plüddemann A Heneghan C Thompson M Wolstenholme J and Price CP Dermoscopy for the diagnosis of melanoma: primary care diagnostic technology update The British Journal of General Practice 2011 61 587 416 417 10.3399/bjgp11X578142 21801535 PMC3103690

[B3] NL Hufford D R. DS Etiological Factors in Skin Cancers: Environmental and Biological In Cancer of the Skin 2010 2 London: Elsevier

[B4] AmericanCancerSociety Cancer Facts&Figures 2010 http://www.cancer.org/acs/groups/content/@nho/documents/document/acspc-024113.pdf Available online (Accessed April 2015)

[B5] CJ Riegel DS Malignant melanoma: prevention, early detection, and treatment in the 21st century CA Cancer J Clin 2000 5 21 10.3322/canjclin.50.4.215 10986965

[B6] AmericanCancerSociety Melanoma Skin Cancer Overview 2010 http://www.cancer.org/acs/groups/cid/documents/webcontent/003063-pdf.pdf Available online (Accessed April 2015)

[B7] Troxel DB Pitfalls in the Diagnosis of Malignant Melanoma: Findings of a Risk management panel study Am J Surg Pathol 2003 27 1278 1283 10.1097/00000478-200309000-00012 12960813

[B8] David E Skin cancer. Melanoma and other specific nonmelanoma skin cancers Cancer 1995 75 S1 245 259 10.1002/1097-0142(19950101)75:1+<245::AID-CNCR2820751310>3.0.CO;2-7 8000999

[B9] Soyer HP Kenet RO Wolf IH Kenet BJ Cerroni L Clinicopathological correlation of pigmented skin lesions using dermoscopy EJD 2000 10 1 22 28 10694293

[B10] Vestergaard ME Macaskill PHPM Holt PE Menzies SW Dermoscopy compared with naked eye examination for the diagnosis of primary melanoma: a meta-analysis of studies performed in a clinical setting British Journal of Dermatology 159 3 669 676 18616769 10.1111/j.1365-2133.2008.08713.x

[B11] Celebi ME Iyatomi H Schaefer G Stoecker WV Lesion border detection in dermoscopy images Comput Med Imaging Graph 2009 33 148 531 Mar 10.1016/j.compmedimag.2008.11.002 19121917 PMC2671195

[B12] Kerri-Ann Norton Iyatomi Hitoshi E Celebi Emre Ishizaki Sumiko Sawada Mizuki Suzaki Reiko Kobayashi Ken Tanaka Masaru Ogawa Koichi Three-phase general border detection method for dermoscopy images using non-uniform illumination correction Skin Research and Technology 2012 18 290 300 10.1111/j.1600-0846.2011.00569.x 22092500

[B13] Celebi ME Kingravi HA and Celiker F Fast colour space transformations using minimax approximations IET Image Processing 2010 4 70 80 10.1049/iet-ipr.2008.0172

[B14] Abbas Qaisar Celebi E. Emre Garcia Irene Fondón Rashid Muhammad Lesion border detection in dermoscopy images using dynamic programming Skin Research and Technology 2011 17 91 100 10.1111/j.1600-0846.2010.00472.x 21226876

[B15] Celebi ME Iyatomi H Schaefer G Stoecker WV Lesion border detection in dermoscopy images Comput Med Imaging Graph 2009 33 148 53 10.1016/j.compmedimag.2008.11.002 19121917 PMC2671195

[B16] Pratt WK Digital image processing PIKS Scientific inside 2007 4 Hoboken, N.J.: Wiley-Interscience

[B17] Gomez D. D Butakoff C Ersboll B. K and Stoecker W Independent histogram pursuit for segmentation of skin lesions IEEE Trans Biomed Eng 2008 55 157 61 Jan 18232357 10.1109/TBME.2007.910651 PMC3161407

[B18] Celebi ME Kingravi HA Iyatomi H Aslandogan YA Stoecker WV Moss RH Malters JM Grichnik JM Marghoob AA Rabinovitz HS and Menzies SW Border detection in dermoscopy images using statistical region merging Skin Res Technol 2008 14 347 53 Aug 10.1111/j.1600-0846.2008.00301.x 19159382 PMC3160669

[B19] Sonka M Hlavac V Boyle R Image processing, analysis, and machine vision 1999 2 PWS publishing Pacific Grove, CA

[B20] Celebi ME Aslandogan YA Stoecker WV Iyatomi H Oka H Chen X Unsupervised border detection in dermoscopy images Skin Res Technol 2007 13 454 62 Nov 10.1111/j.1600-0846.2007.00251.x 17908199 PMC3191533

[B21] Celebi ME Wen Q Hwang S Iyatomi H Schaefer G Lesion Border Detection in Dermoscopy Images Using Ensembles of Thresholding Methods Skin Res Technol 2013 19 1 e252 8 10.1111/j.1600-0846.2012.00636.x 22676490

[B22] Mete M Sirakov NM Lesion detection in demoscopy images with novel density-based and active contour approaches BMC Bioinformatics 2010 11 S23 20946607 10.1186/1471-2105-11-S6-S23 PMC3026371

[B23] Peruch F Bogo F Bonazza M Cappelleri VM Peserico E Simpler, Faster, More Accurate Melanocytic Lesion Segmentation Through MEDS IEEE Transactions on Biomedical Engineering 2014 61 2 557 565 24081839 10.1109/TBME.2013.2283803

[B24] Martin Ester Kriegel Hans-Peter Sander J Xu Xiaowe A density-based algorithm for discovering clusters in large spatial databases with noise Proceedings of 2nd International Conference on Knowledge Discovery and Data Mining (KDD-96) 1996 226 231

[B25] Caihong Yang Wang Fei Huang Benxiong Internet traffic classification using DBSCAN Information Engineering, 2009 2009 ICIE'09. WASE International Conference 163 166

[B26] John Rushing Tiller John McDowell Drew Tanner Steve Adaptive Artificial Intelligence for Next Generation Conflict Final Tech Report, TILLER (JOHN) MADISON AL 2004

[B27] Xueping Su Peng Jinye Feng Xiaoyi Wu Jun Fan Jianping Linking names and faces by person-based subset clustering Proceedings of the Third International Conference on Internet Multimedia Computing and Service 2011 120 123

[B28] Mete M Tang F Xu X Yuruk N Finding Functional Modules In Systems Biology for Signaling Networks 2010 Springer New York 253 273

[B29] Mete M Tang F Xu X Yuruk N A structural approach for finding functional modules from large biological networks BMC Bioinformatics 2008 9 S19 18793464 10.1186/1471-2105-9-S9-S19 PMC2537570

[B30] Ertoz L Steinbach M Kumar V Finding Clusters of Different Sizes, Shapes, and Densities in Noisy, High Dimensional Data In Proceedings of Second SIAM International Conference on Data Mining 2003

[B31] Mete M Kockara S Aydin K Fast density-based lesion detection in dermoscopy images Comp Med Imag and Graph 2011 35 2 128 136 10.1016/j.compmedimag.2010.07.007 20800995

[B32] Tasneem Brutch Bourges-Sevenier Mikael Khronos WebCL: Accelerating Web Applications 2013

[B33] David Luebke Harris Mark Govindaraju Naga Lefohn Aaron Houston Mike Owens John Segal Mark Papakipos Matthew Buck Ian GPGPU: general-purpose computation on graphics hardware Proceedings of the 2006 ACM/IEEE conference on Supercomputing, ACM 2006 208

[B34] Jeon Won Gibbs Simon WebCL for Hardware-Accelerated Web Applications Tizen Developer Conference 2012

[B35] Erik Smistad Elster Anne C Lindseth Frank Fast Surface Extraction and Visualization of Medical Images using OpenCL and GPUs The Joint Workshop on High Performance and Distributed Computing for Medical Imaging 2011

[B36] Jeon W Brutch T Gibbs S WebCL for hardware-accelerated web applications WWW'12 Dev 2012 Lyon, France

[B37] Massoud Pedram Brooks David Pinkston Timothy Report for the NSF Workshop on Cross-layer Power Optimization and Management NSF Workshop on Cross-Layer Power Optimization and Management 2012

[B38] Stone John E Gohara David Shi Guochun OpenCL: A parallel programming standard for heterogeneous computing systems Computing in science & engineering 2010 12 3 66 21037981 10.1109/MCSE.2010.69 PMC2964860

[B39] Munshi Aaftab The OpenCL Specification Khronos OpenCL Working Group 2011 1.1 doc. revision 44

[B40] Brutch Tasneem WebCL Overview Roadmap DevCon 5 2011

[B41] Wang Jigang and Cooper Leon N Improving nearest neighbor rule with a simple adaptive distance measure Pattern Recognition Letters 2007 28 2 207 213 10.1016/j.patrec.2006.07.002

[B42] Amdahl GM Validity of the single processor approach to achieving large scale computing capabilities AFIPS spring joint computer conference 1967 483 485

[B43] Schaefer G Rajab MI Celebi ME Iyatomi H Colour and contrast enhancement for improved skin lesion segmentation Computerized Medical Imaging and Graphics 2011 35 99 104 10.1016/j.compmedimag.2010.08.004 21035303

